# Videos

**DOI:** 10.1155/1993/37231

**Published:** 1993


### HEMIHEPATIC VASCULAR OCCLUSION DURING RESECTION OF SEGMENTS III AND IV FOR HCC IN CIRRHOTIC LIVER

R Dionigi, L Dominioni, G Carcano, A Benevento, S Cuffari, R Galozzi

Department of Surgery, University of Pavia-Varese, Ospedale Multizonale di Varese, Italy

In the cirrhotic liver with deep-seated multifocal hepatocellular carcinoma (HCC) hepatic resection should be carried out in anatomical planes, to avoid extensive bleeding and leave sufficient functional parenchyma. However the cirrhotic liver poorly tolerates complete liver ischemia, and every effort should be made to minimize the duration and extension of total hepatic vascular occlusion.

CASE REPORT. The case of a 55-year-old male patient with a history of alcoholic cirrhosis, portal hypertension and with histologically proven chronic active hepatitis is reported. During follow up with liver ultrasound monitoring he was discovered to have hyperechogenic nodules in liver segment III (12mm diameter) and segment IV (25mm diameter). Needle biopsy of the nodules showed HCC; alphafetoprotein plasma level was high: 50 ng/dl. The patient underwent initially arterial chemoembolization with lipiodol-epirubicin and then was proposed for surgical resection of the bifocal neoplasia. The surgical treatment consisted of combined resection of segments III + IV. We used the new hepatectomy method described by Makuuchi and coworkers, based on intermittent hemihepatic vascular occlusion, selective segmental devascularization and ultrasonically guided liver resection. This video describes the technique used. Intermittent hemihepatic vascular occlusion (15 min. ischemia interrupted by 5 min. reperfusion) allowed to operate in conditions of ischemia firstly of the left lobe, during segment III dissection, and subsequently of the right lobe, during segment IV dissection. Intraoperative ultrasonography permitted the recognition of the two neoplastic foci, of the portal tributaries to segment IV (P4) and of two portal branches for segment III (P3A, P3B). Under ultrasound control, indigocarmine dye was injected through transhepatic puncture in P4, P3A and P3B at their origin to demarcate the parenchyma to be resected. Segments III and IV were then resected with preservation of the vasculature of all residual segments.

RESULTS. Total left liver ischemia was 42 min. and right liver ischemia was 47 min. The technique of intermittent hemihepatic vascular occlusion allowed uncomplicated and oncologically radical bisegmentectomy III + IV in a cirrhotic liver, with low intraoperative blood loss (900 ml) not requiring hemotransfusion and without subsequent liver insufficiency. The postoperative course was uncomplicated and the patient was discharged after 15 days.

VF002

### SIMPLE CYST OF THE LIVER LAPAROSCOPIC MANAGEMENT

J M Jover, J C R Adana, J Lopez, M Limones,
J L Ramos, P Artunedo, M Moreno

Hospital Universitario de Getafe, Madrid,
Spain

Simple cysts of the liver are cystic formations containing a serous fluid, not communicating with the intrahepatic biliary tree. In most cases, simple cysts are asymptomatic and remain silent. The treatment of choice for symptomatic or complicated simple cyst is partial excision of the external part of the cyst.

This video shows the laparoscopic treatment of a liver cyst. A 45 year old woman with abdominal pain and discomfort was treated by percutaneous aspiration but the symptomatology returned after two months and other causes of the symptoms were excluded. The diagnosis was completed by ECO and TAC.

Under general anaesthesia laparoscopy was performed with C02 insufflation using a 10 mm end-viewing introduced through a disposable spring-loaded 10 mm trochar and cannula and inserted through an infra-umbilical incision. The cyst was identified in the dome of the right liver. Further access to the peritoneum was gained by inserting a second 10 mm and two 5 mm ports. The cyst content was aspirated and the roof of the cyst was excised by diathermy.

The patient was discharged home on the second post operative day. Histology confirmed the benign nature of the cyst wall.

Laparoscopic excision of the cyst roof reduces the morbidity and hospital stay provides excellent post operative comfort and avoids a bilateral subcostal incision.

VF003

### RIGHT HEPATECTOMY AND RESECTION OF INFERIOR VENA CAVA WITH VASCULAR EXCLUSION

G Gozzetti, A Mazziotti, E Jovine, GL Grazi, A Frena, F Pierangeli

2° Dpt. of Surgery, Univ. of Bologna, S. Orsola Hospital, Bologna, ITALY

Total vascular exclusion (TVE) has been proposed for hepatic tumors located in the posterior segments of the liver, strictly adjacent or infiltrating the vena cava. The video shows the case of a 48 year old patient operated on 4 years before of partial colectomy for cancer and 2 years later of right adrenectomy for metastases. Follow-up echography and subsequent CT scan revealed the presence of a recurrence in the right adrenal space, infiltrating the liver, the kidney and closely adherent to the retrohepatic vena cava, without signs of intraluminal diffusion at cavography. A conventional TVE has been carried out, clamping the subhepatic vena cava just above the renal veins, the hepatic peduncle and the sovrahepatic vena cava. Then, the right hepatectomy has been accomplished and the retrohepatic vena cava has been freed from the caudate lobe; at that time a 2 centimeters infiltration of the middle part of the vena cava wall became evident. After having sutured the right hepatic vein stump, the upper vena cava clamp has been moved below the confluence of the middle and left hepatic veins, leaving in place the caval clamp above the renal veins. The clamp at the hepatic hilum has been removed and the remaining liver is revascularized after 40 minutes of TVE. The vena cava has been resected and removed with the right emi-liver; the specimen confirms the tumoral infiltration of the vena cava layer. Vena cava reconstruction has been carried out with prosthetic Dacron graft. The operation has been completed with the right nefrectomy. The angio-CT control performed 2 weeks later showed the patency of the caval graft.

VF004 HEPATOTOM SUPERSONIC MICROJET DISSECTOR IN HEPATIC RESECTION

G J Poston

Surgical Oncology Unit, Royal Liverpool
University Hospital, Liverpool, UK.

Resection is now the established treatment in many cases of primary and secondary hepatic tumors. Operative mortality rates should not exceed 2%, and operative morbidity (particularly biliary leaks) should be minimal. Baer and Blumgart have developed the Hepatotom supersonic microjet dissector to facilitate hepatic resection with minimal bleeding and maximal exposure of blood vessels. The purpose of this presentation is to demonstrate its use in my hands and review my early experience using the Hepatotom.

The video demonstrates the complete resection of a tumor arising in segment 7 using the Hepatotom. This is performed without the aid of portal cross clamping and using only standard monopolar diathermy for cautery. Total operative blood loss was 600 ml, and the patient was discharged from hospital on the 6th postoperative day.

Using the Hepatotom, I have now resected 14 hepatic tumors (13 colorectal metastases and 1 FNH) from 8 patients, median age 68 (range 43-80) years. Median blood loss per resection was 400 (50-1000) mls, and median postoperative stay was 8 (6-17) days.

I conclude that the Hepatotom is a major advance in hepatic resectional surgery and further trials are now necessary to compare its efficiency with other resectional techniques in liver surgery.

VF005 **DEFINITIVE SURGICAL TREATMENT OF COMPLICATED HYDATID CYST OF THE LIVER**

**E Moreno González, A De La Calle, P Rico, P Vorwald, V Maffettone, A López**

***Department of General and Digestive Surgery - Unit of Liver Transplantation, Hospital "12 de Octubre", Complutense University of Madrid, Madrid, Spain***

**The patient included in this film was operated on eigth times because he suffered from giant hydatid cyst of the right lobe of the liver. The first operation was marsupialization, producing permanent fistula and infection. The second: partial resection and drainage. The third, sphincteroplasty, The fourth: opening of the residual cavity, drainage and choledocotomy plus external drainage. The other three operations were performed by percutaneous approach by interventional radiology (dilatation of the biliary branchs and transtenotic stent). The patient continued with cholangitis, obstructive jaundice and external fistulae. The operation was performed through a right subcostal incision. Right duct and confluence were obstructed. The right portal vein was thrombosed. The right lobe of the liver was atrophic. Right lobectomy extended to the biliary confluence was performed. Left hepatic duct-jejunal anastomosis (side-to-side) was done. The postoperative period was uneventful. The patient is asymptomatic two years after the operation.**

VF006

### LIVER AND KIDNEY TRANSPLANTATION FOR CHRONIC HAEMODYALISIS PATIENT WITH LIVER INSUFFICIENCY

**E Moreno González, I García, C Loinaz, I G-Pinto, R Gómez, R Jiménez, V Maffettone, J Ibáñez.**

*Department of General and Digestive Surgery - Unit of Liver Transplantation, Hospital "12 de Octubre", Complutense University of Madrid, Madrid, Spain*

The patient included in this film suffered from chronic renal insufficiency, being included in a haemodyalisis programm. Two years later a kidney transplant was performed and untreatable acute rejection occurred. The graft was removed and the patient went back to haemodyalisis for seven years. Contamination by HCV and cirrhosis occurred with evolution to liver insufficiency. Evaluation of the functional activity of the liver was done and a synchronous liver and kidney transplantation was performed. The film shows the different steps of the double organ transplantation, during the total hepatectomy, vascular and biliary anastomosis of the liver graft and later the kidney. The film finaly shows the inmediate aspect of the patient and six months later. The postoperative course of the patient was completely normal.

VF007

### LIVER TRANSPLANTATION WITH PRESERVATION OF RECIPIENT RETROHEPATIC VENA CAVA

G Gozzetti, A Mazziotti, E Jovine, GL Grazi, A Frena, M Morganti

2° Dpt. of Surgery, Univ. of Bologna, S. Orsola Hospital, Bologna, ITALY

Clamping of the vena cava (VC) and of the portal vein during the conventional orthotopic liver transplantation (OLT) technique, causes important hemodynamic consequences and oblige to the use of the veno-venous by-pass in the majority of the cases. Furthermore freeing the retrohepatic VC can bring to hemorrhage from the adrenal space, often difficult to control. Reported initially for pediatric OLTs, the hepatectomy with VC preservation technique avoids the total clamping of the VC, therefore the need of veno-venous by-pass, and lowers the hemorrhagic risk due to the dissection of the retrohepatic VC. The technique has been recently modified to be applied in adults with same purposes. The movie shows OLT in an adult patient with post-necrotic liver cirrhosis. Mobilization of the liver and freeing of the hepatic peduncle have been carried out with the standard technique. The accessory hepatic veins have been dissected and cutted to expose the anterior portion of the VC, avoiding to dissect its lateral portions. A temporary porto-caval T-L anastomosis has been carried out with the right branch of the portal vein. Hepatectomy has been completed after the clamping and suturing of the main hepatic veins. The recipient VC has been laterally clamped to maintain a blood flow and the graft upper VC has been anastomized termino-laterally just below the recipient hepatic veins. Subhepatic graft VC has been stapled after having flushed the liver with a 5% albumin solution. At this time, the porto-caval anastomosis has been removed, the VC wall sutured and the recipient portal vein anastomized with the graft portal vein. No modifications have been seen in the hemodynamic status of the patient while performing vascular anastomoses.

VF008

### LIVER HEMANGIOMAS. ABSTENTION, RESECTION, TRANSPLANTATION

G Gozzetti, A Mazziotti, E Jovine, GL Grazi, A Frena, A Gallucci, M Masetti, G Ercolani

2° Dpt. of Surgery, Univ. of Bologna, S. Orsola Hospital, Bologna, ITALY

This video sums up the ten year experience of Clinica Chirurgica 2° of the University of Bologna on liver hemangiomas. The clinical series is composed of 77 cases of "giant" hemangioma: 27 underwent a liver resection (6.5% of all liver resection performed during the same period), 1 patient was transplanted and another with the liver completely replaced by an hemangioma with significant distress is in waiting list for a transplantation. In 48 patient with large but stable asymptomatic hemangiomas, a wait and see policy was adopted. Spontaneous hemorrhage was never observed. Since these lesions are benign, surgery should be considered only in the presence of symptoms or enlargement. Liver transplantation should be considered when the hepatic parenchyma has been completely replaced in highly symptomatic patients. The video presents the main stages of a major hepatectomy, a wedge resection and a liver transplantation without veno-venous by-pass.

VF009

### Pediatric liver transplantation from related living donor

Boillot O, Dawahra M, Porcheron J, Houssin D, Voiglio E, Cloix P, Boucaud C, Stamm D, Gille D, Bodnar D, Mion F, Kopp C.
Unité de Transplantation Hépatique, Pavillon V, Hôpital E. Herriot, Place d'Arsonval, 69003 Lyon, France.

Pedriatic liver transplantation now represents about 15% of liver transplantation (LT) performed in France. In spite of the use of reduced sized and split liver grafts, some children still die before a cadaveric liver can be available for LT.

The concept of liver transplantation from a living related donor arose from several reasons:
1) the need to increase a stagnant number of cadaveric donors, while the number of adult and pediatric candidates to liver transplantation is steadily increasing;
2) the improvments of hepatic surgery allowing a very low mortality rate after hepatectomy in non cirrhotic patients;
3) the conclusive results of liver transplantation obtained with reduced size livers and split liver, appearing as a preliminary step to the technique of the living donor;
4) the increased immune compatibility represented by the living related donor.

On july 22, 1992, we underwent our first pediatric liver transplantation from a living donor.

**Patients and methods.** The diagnosis of biliairy atresia was established in a 6 months old female, when she developed ascitis and icterus. At this time, a Kasai operation was not performed. Echography with Doppler examination showed a patent portal vein with hepatopetal blood flow and normal diameter (6 mm). Two months later, the conditions of the child were worsering with intractable ascitis, increasing jaundice and stagnation of the body weight at 7,6 kg despite continous enteral nutrition.

The father, an healthy 27 years old man, asked us to be a volonteer as donor. The concept of this innovative therapy had been already submitted to a research-ethics consultation which gave us favorable conclusions. The father and her child were of the same blood type O Rhesus positive, and shared 4 MHC antigens. After donor carefull psychological, clinical, biological and radiological evaluations, the left lateral hepatic lobe (segments II and III) was harvested, taking away left hepatic artery, left portal vein and left hepatic vein ; hepatic artey for segment IV, which arised from the right arterial structures, was preserved. Graft cold ischemia time in UW solution was 1 hour and 45 minutes. The graft was orthotopically transplanted, after total recipient hepatectomy with inferior vena cava preservation. Revascularization of the graft was homogeneous from the very beginning and its early function was excellent.

**Results.** Thirdteen days after the operation, the donor was discharged in good conditions. The child was reoperated at day 9 for a small biliary leak originating from the cutted surface of the liver. After medical resolution of a rejection episode and an abdominal infection, the child was discharged in good health with nomal liver function and patent hepatic vessels, one month post-transplant.

**Discussion.** This case report confirms the feasibility of pediatric liver transplantation from a living related donor, as it has been previously reported by others. The surgical risk was judged to be acceptable for a parent volunteering to become an organ donor, with regard to the benefits expected for the child. A complete morphological evaluation (CT scan and celiac angiography of the donor liver appears mandatory, in order for the surgeon to know exactly beforehand if hepartectomy is feasible and which type can be performed.

With regard to ethical problems, the concept of living related donor sounds reasonable, as long as the donor volunteers without outside pressures and with complete knowledge of the risks and the possible therapeutic alternatives offered to his or her child. This technique may increase the pool of potential grafts, although it won't be sufficient to overcome the present shortage of organs. In any case, this technique should: 1) provide excellent liver grafts; 2)decrease the cold ischemia time greatly; 3) allow the liver transplantation to be performed electively, without any potentially damaging delay for the recipient.

VF010

### A POSTERIOR INTRAHEPATIC APPROACH TO THE GLISSONIAN SHEATHS, FOR LIVER RESECTION

Bernard LAUNOIS, Glyn G. JAMIESON

Centre for Digestive Surgery, Hospital Pontchaillou and Department of Surgery Royal Adelaïde Hospital Adelaïde

For liver resections, the structures of the hepatic trinity can be approached either outside or within the liver substance. We have developed a new approach which we believe combines and extends the advantages of the other approaches. An incision is made immediately anterior to the hilum and a second incision is made in the caudate process immediately posterior to the hilum. The surgeons finger and thumb are insinuated through these incisions into liver substance and the confluence of the main sheaths is dissected. This gives immediate access to the main right and left sheaths. For segmental resections from the right side of the liver several peripheral sheaths can be dissected out by this approach. Thus a sheath to segment VI (and sometimes the main right lateral sheath to segments VI and VII) and the main sheath to segments V and VIII can almost always be dissected free. Clamping of the sheaths, with consequent colour change in the liver, allows early delineation of the main fissure and more importantly the right fissure. The approach is useful also in patients with high bile duct cancer, for determining the proximal extent of the cancer and for providing access to the right and left hepatic ducts, if a biliary enteric bypass is chosen. We believe this posterior approach to the Glissonian sheaths is an extremely useful addition to the liver surgeon's armamentarium.

VF011

### NEW TECHNOLOGIES IN LAPAROSCOPIC CHOLECYSTECTOMY: ULTRASONOGRAPHY, CUSA AND ARGON LASER

A Benevento, G Carcano, S Cuffari, P F Interdonato, M Bianchi, R Dionigi

Department of Surgery, University of Pavia-varese, Ospedale Multizonale di Varese, Italy

Laparoscopic cholecystectomy represents the " gold standard" for treatment of symptomatic gallstones and gallbladder polyps. Increasing experience with this technique has broadened the indications to include acute cholecystitis and chronic sclerotic cholecystitis. In such conditions pathologic modifications of the anatomy of the biliary tree could enhance the risk of lesion of the common bile duct during isolation of the cystic duct or the risk of bleeding from the hepatic bed or from an unidentified cystic artery.
In performing simple cholecystectomy the presence of anatomical variations of the biliary and arterial trees and the possibility of choledochal gallstones, eventually descended from the cystic duct during laparoscopic manoeuvres, should be considered. Laparoscopic cholangiography is not widely performed for the potential risk of further choledochal lesions and for the long time usually required to perform this procedure.

The video shows the use and the reliability of the laparoscopic 10 MHz Aloka probe in performing safely and quickly an ultrasonography of the triangle of Calot and of the hepato-duodenal ligament during laparoscopic cholecystectomy. Cystic duct, cystic artery and common bile duct are evaluated before and after gallbladder removal, in order to exclude potential hazard to the biliary tree and to verify the absence of residual choledochal stones.
The CUSA dissector facilitates the careful anatomical preparation of the cystic duct and of the cystic artery. Accurate dissection of the gallbladder from the hepatic bed is also performed with this probe that accurately spares even minimal vessels.
The Argon laser is then used to allow a safer hemostasis of the liver surface.

The effective cost of this tecnique is overcome by the absence of even minimal bleeding and by the safety in sparing the hazard of biliary tree lesions.

VF012 **RESECTION OF BILIARY MALIGNANCIES AFTER INTERVENTIONAL RADIOLOGY TREATMENT**

**E Moreno González, R Gómez, I García, J Ibáñez, JC Palomo, V Maffettone, D Hernández**

***Department of General and Digestive Surgery - Unit of Liver Transplantation, Hospital "12 de Octubre", Complutense University of Madrid, Madrid, Spain***

This film includes the definitive operation on a patient suffering of obstructive jaundice, produced by malignant tumour previously treated by percutaneous intallation of a expander prosthesis. A subcostal incision was made. The hepatoduodenal ligament was explored demonstrating absence of extrahepatic malignancies. Portal vein, common hepatic artery and choledocus were dissected distally, removing the biliary tract and all lymphaty tissue around it, in one block with the gallbladder and the distal part of the two paramedian segments. In the upper end the biliary tract was removed with the segmental branches, taking a free margin of more than 15 mm. In order to perform a correct anastomosis the segmental branches in the right and in the left lobe were anastomosed obtaining two wide stomas, right and left which were anastomosed separately to the proximal end of a Roux-en-Y jejunal loop. The movie finishes showing the specimen which include the expander in side partially obstructed.

VF013

### LEFT HEPATICO-GASTROSTOMY UNDER LAPAROSCOPIC GUIDANCE FOR MALIGNANT BILIARY OBSTRUCTION.

M. Gagner, G. Soulez, E. Deslandres,
R. Leduc, A. Pomp
Departement of Surgery, Radiology and
Gastroenterology, Hotel-Dieu de Montreal,
University of Montreal
Montreal, Quebec, Canada

Laparoscopic palliative procedures were performed for relief of jaundice due to unresectable peri-ampullary tumors. Cholecystoenterostomy is presently being evaluated by several institutions, but may lead to failures due to malignant encasement of the cystic duct.

We have developped a new technique of biliary decompression using segment III bile duct with the lesser curvature of the stomach. The bile duct is access under fluoroscopic guidance, the guide wire introduced is perforated under segment III of the liver, and guided laparoscopically in the lesser curvature of the stomach. With peroperative gastroscopy the wire is removed through the mouth where a percutaneous endoscopic gastrostomy (PEG) tube is introduced. This is placed into the stomach and biliary tree in a retrograde manner. A fibrin glue is used to seal the hepatico-gastric surfaces. The tube is removed 2 weeks later, leaving a permanent hepaticogastric fistula.

Three patients were treated with a follow up of 11 months. One cholangitis occur in the post-op period. One patient had a liver capsule hemorrhage which necessitated a blood transfusion. All patients have been palliated satisfactorily. One patient died 9 months later of metastatic pancreatic carcinoma with an intact fistula at autopsy. This method is an alternative to the classical cholecysto or hepaticoenterostomy.

VF014 HETEROTOPIC LIVER TRANSPLANTATION IN THE RAT.

M.A. Aller, L. Lorente, J. Rodriguez, S. Alonso, J.I. Trobo, J. Arias

Surgery I Dept. Complutense University of Madrid. Spain

Heterotopic or auxiliary liver transplantation (ALT) is an attractive alternative to the orthotopic liver transplantation and provide temporary support of a potentially reversible liver damage. One cause of the transplant failure is the inter-liver competition of the host liver and the graft for the use of hepatotrophic factors found in the portal blood. We have developed an experimental model of heterotopic partial liver isotransplant in the rat using a microsurgical technique so as to study the inter-liver competition.
The auxiliary liver graft consists of the right lateral and caudate lobes. The donor hepatectomies reduce the graft mass, facilitate the recipient operation and diminish the complications secondary to the graft's revascularization. The cuff technique for the portal vein anastomosis not only simplifies the microvascular anastomosis but also shortens the portal hypertension in the recipient from an average of 15 minutes when the suture anastomosis is used to approximately seven minutes when our technique is used. The venous drainage by the infrahepatic inferior vena cava prevented the graft congestion and increased the post-operative survival rate that had occurred when we used the suprahepatic venous drainage. The bile duct was passed into the duodenum of the recipient and fixed to the wall by a simple stitch.
The graft liver was vascularized with blood from the portal vein and the liver of the recipient was only vascularized arterially.
As the graft underwent regeneration during the first 30 days of the postoperative period, it may be considered that the partial heterotopic transplant of the liver supports the hepatic function.

VF015

### PANCREATIC DUCTAL STONES. LASER LITHOTRIPSY AND WIRSUNGOGASTRIC ANASTOMOSIS

J L Gouzi, E Bloom, Ch Julio, B Pradere

CHU Purpan, Place du Docteur BAYLAC, 31059 Toulouse Cedex, France

This video reports the record of a 36 year old patient presenting with chronic pancreatitis.

The diagnosis of obstructive ductal stone is assessed in the course of an attack of benign acute pancreatitis. The ductal stone is localized by ultrasound and CT scan, showing an impaction of a 1 cm stone in the isthmic segment of the Wirsung along with a dilatation of the pancreatic duct caudad. Several other smaller stones are found in both cephalic and caudal pancreas.

The anterior aspect of the pancreas is approached after separation of the omentum from the transverse colon. The pancreas is incised longitudinally over the dilated duct and the impacted stone, which is removed with forceps. An ureteroscope is inserted into the pancreatic duct cephalad and caudad.

A flash lamp excited dye laser is introduced in the operative channel of the ureteroscope, allowing visual control of targeting the laser fibre onto the stone surface. Laser lithotripsy is continued to obtain both ductal clearance and a free transpapillary passage. Ductal lithotripsy is completed by a long laterolateral Wirsungogastric anastomosis.

The permeability of the cephalic duct and of the Wirsungogastric anastomosis is controlled endoscopically on the 30th post operative day.

This ductal clearance technique provides a simple "anatomical" surgical solution to young patients suffering with chronic pain, thus avoiding pancreatic resection.

VF016

### HAEMOSUCCUS PANCREATICUS: RESULTS OF ARTERIAL EMBOLIZATION AND SURGICAL TREATMENT

P Petrin, M Antoniutti, D Zaramella,
C Da Lio, V Costantino, S Pedrazzoli

Istituto di Semeiotica Chirurgica, Ospedale
CTO, University of Padova, Italy

Bleeding from the pancreatic duct is a difficult to diagnose, life-threatening complication of chronic pancreatitis (CP).

Among 220 treated CP: 10 had one or more intestinal haemorrhages from the pancreas: mean age was 47 years, male/female ratio 7/3. Pancreatic psuedocysts were present in 6 and calculi in 5. The bleeding was single and massive in 5 (Hb below 8gm/dl), slow and recurrent in 5. Diagnosis was made by means of selective arteriography in 7 (the bleeding pseudoaneurysmatic artery was: pancreatico-duodenal in 3, splenic in 2, superior mesenteric in 1); suspected on the basis of scintigraphy with labelled RBC in 1 (arteriography was negative); of contrast-enhanced CT scan in 1. In 1 case the diagnosis was made at surgery. Just in 4 out of 9 cases EGDS were positive. 4 patients underwent artery emoblization and 3 days later one of them had emergency left pancreatectomy. Another one died 8 years later from massive intestinal bleeding. 6 underwent straight surgery: 2 had left pancreatectomy (1 died 2 years later from recurrence), 2 pancreato-duodenectomy, 2 pancreatico-jejunostomy and surgical haemostasis. Even after embolization or resective surgery recurrence of bleeding from the pancreatic duct can be possible as well as fatal.

VF017

### MODIFICATION OF THE WHIPPLE PROCEDURE: PANCREATIC HEAD RESECTION WITH PYLORUS PRESERVATION AND PANCREATICOGASTROSTOMY

L Harsányi, T Tihanyi, L Flautner

1st Surgical Department, Semmelweis Medical School, Budapest, Hungary

Pancreatoduodenectomy became a routine procedure for the treatment of malignancies of the pancreatic head, papilla of Vater, duodenum and distal choledochus as well as for chronic pancreatitis localised to the pancreatic head region. During the last fifty years several modifications of the original Whipple technique were developed in order to decrease operative mortality and morbidity. Based upon early and late results this technique developed at our clinic proved to be the method of choice in our experience. The surgical procedure is shown in this video presentation.

VF018

### "INTERMEDIATE PANCREATECTOMY" AS RESECTION OF BENIGN TUMORS LOCATED IN THE NECK OF THE PANCREAS.

G. Serio, C. Iacono, G. Mangiante, F. Nifosì, N. Cracco, . Zanza.

Department of Surgery, Division of General Surgery "C", Verona University, Verona, Italy.

When dealing with benign pancreatic tumors located between head and body, 2 or more cm. in size, we have to take into account some technical and biological considerations. Enucleoresection can damage the main duct. Resection of body and tail, plus splenectomy, could be another surgical solution, but this procedure may lead to permanent impairment of the glycoregulation.

On the other hand duodenopancreatectomy portends high morbidity and non negligible operative mortality and carries various degrees of digestive function derangement. Moreover both types of resection-notably the latter- are definitely disproportionate in the treatment of benign or mainly benign tumors. We believe that a segmental resection, performed at least one centimeter from the tumor margins, can be aimed at saving uninvolved parenchyma and at performing an oncologically radical operation, also in cases of islet cell tumors or mucinous cystoadenomas,neoplasms of uncertain benign nature. Following a midline incision the anterior pancreatic surface is exposed; the posterior sheet of peritoneum is cut along the lower and upper edge of the pancreatic segment concerned. The splenic vein and when necessary the artery, are dissected free from the posteroir aspect of the gland, and the involved pancreatic segment is resected with at least 1 centimeter of free margins from the neoplasm (fig. A). The distal stump is subjected to end-to-end anastomosis with a telescopic double-layer Roux - en Y jejunal loop, whereas the cephalic stump is sutured with interrupted stitches after elective ligature of the main duct (fig. B). Nine patients were given this procedure ( 3 insulinomas, 4 serous cystadenomas, 1 mucinous cystadenoma, 1 non functioning cystic islet tumor ). At follow - up ranging from 8 months to 7 years the glycoregulation tests were normal, and no recurrence was observed. In conclusion intermediate pancreatectomy proved to be higly reliable as regard both operative and oncological considerations.

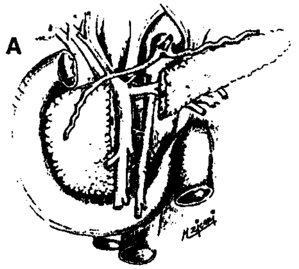

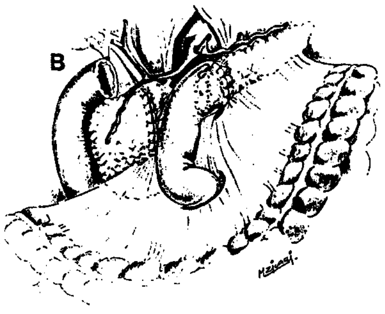

VF019

### DIAGNOSIS AND SURGICAL TREATMENTS OF INSULINOMAS.

G. Serio, C. Iacono, G. Mangiante, F. Nifosì, N. Cracco, C. Benassuti.

Department of Surgery, Division of General Surgery "C", Verona University, Verona, Italy.

Insulinomas still present difficult problems as regards their clinical diagnosis, precise localization and choice of the most appropriate type of resective surgery. Clinical confirmation is based on detection of plasmatic hyperconcentration of insulin and / or C-Peptide, and on the clinical response to inhibition / stimulation tests.
The essential step in planning surgical treatment is however morphological and topographical identification, which is allowed in about 80-90% of cases by modern imaging techniques ( U.S., C.T., Angiography, venous sampling ). The diagnostic work - up is completed by the intraoperative exploration of the gland by means of careful palpation and I.O. ultrasonography.
Due to the benign nature of the tumors ( roughly in 90% of cases ) and small size, pancreatico-duòdenectomy and even distal pancreatectomy appear out of proportion, requiring a too wide tissue demolition.
On the other hand simple enucleation is fraught with oncological and technical reservations; such a procedure is therefore well fitted only for cephalic and, to a lesser extent, for superficial tumors in the body-tail measuring less then 15 mm in diameter. For lesions in the neck or contiguous tract of the head and body with diameters close to or exceeding 2 cm, or lesions extending deeply into the gland, intermediate pancreatectomy appears to be the best tailored surgical approach, coupling the need for radicality with the desire to save parenchyma.
When faced with tail and distal body lesions, mostly those more than 1 cm in size and deep lying, we prefer opting for distal pancreatectomy, almost invariably performed with spleen preservation. In the occurrence of unlocalized insulinoma we adopt a wait and see type of management, always avoiding blind subtotal pancreatectomy; the endocrine syndrome is controlled pharmacologically and the patient is regularly followed by U.S. or C.T.

VF020

### LEFT PANCREATECTOMY AND PRESERVATION OF THE SPLEEN FOR TRAUMATIC RUPTURE OF THE NECK OF THE PANCREAS

C.H JULIO, E. BLOOM, F. DE HALDAT, J.L GOUZI

C.H.U PURPAN - Place du Dr BAYLAC
31059 - TOULOUSE Cedex - FRANCE

This video reports an original technique of left pancreatectomy preserving the spleen performed as an emergency procedure to treat a traumatic rupture of the neck of the pancreas.
The lesional diagnosis was assessed by ultrasound and CT scan early after a blunt abdominal trauma. Surgery was performed within the 8th post traumatic hour.
The anterior aspect of the pancreas is exposed by separating the omentum from the colon taking much care not to harm the short gastric vessels.
The complete rupture of the neck of the pancreas is displayed after debridment of the traumatic hematoma, thus freeing the anterior aspect of the mesenteric vein.
A left pancreatectomy is performed after mobilization of the tail of the pancreas. Splenic vessels are ligated and divided proximal to the splenic hilum, thus allowing conservation of the spleen. The remaining blood flow to the spleen originates from short gastric, gastroepiploic and left gastric arteries.
Arteriography and scintigraphy on the second post operative month reveal a good splenic injection with a complete parenchymography.
This technique of left pancreatectomy with resection of the splenic pedicle has already been described for benign tumors and pancreas donation from a living relative. Preservation of the spleen is possible in emergency procedures.

VF021

### EARLY EXPERIENCE WITH LAPAROSCOPIC PANCREATODUODENECTOMY.

M. Gagner, A. Pomp, C. Potvin, E. Deslandres

Department of Surgery, Hotel-Dieu de Montreal,
University of Montreal
Montreal, Quebec, Canada

Diagnostic laparoscopy is usefull to exclude metastasis from pancreatic or periampullary neoplasms. We have performed two laparoscopic pylorus preserving pancreatoduodenectomy in two patients: A 33 year old female with chronic pancreatitis and pancreas divisum and a 75 year old female with an adenocarcinoma of the ampulla of Vater. We have used five 11 mm trocars in the right upper quadrant and one 18 cm umbilical trocar.

An endoscopic linear cutter 60 mm long was used to transect the duodenum, 1 cm distal to the pylorus and at the jujunum junction. The resected specimen was extracted in a sterile plastic bag. The postoperative stay was prolonged (30 and 62 days) because of a delay in gastric emptying and a small pancreatic fistula. Although this series is too small to draw adequate conclusions, we feel that it is technically possible but may not be desirable for the patient due to the complexity of the surgery.

